# The left ventral premotor cortex is involved in hand shaping for intransitive gestures: evidence from a two-person imitation experiment

**DOI:** 10.1098/rsos.181356

**Published:** 2018-10-10

**Authors:** Arran T. Reader, Nicholas P. Holmes

**Affiliations:** 1 Department of Neuroscience, Karolinska Institutet, Stockholm, Sweden; 2 Centre for Integrative Neuroscience and Neurodynamics, School of Psychology and Clinical Language Sciences, University of Reading, Reading, UK; 3 School of Psychology, University of Nottingham, Nottingham, UK

**Keywords:** motion-tracking, motor control, social interaction

## Abstract

The ventral premotor cortex (PMv) is involved in grasping and object manipulation, while the dorsal premotor cortex (PMd) has been suggested to play a role in reaching and action selection. These areas have also been associated with action imitation, but their relative roles in different types of action imitation are unclear. We examined the role of the left PMv and PMd in meaningful and meaningless action imitation by using repetitive transcranial magnetic stimulation (rTMS). Participants imitated meaningful and meaningless actions performed by a confederate actor while both individuals were motion-tracked. rTMS was applied over the left PMv, left PMd or a vertex control site during action observation or imitation. Digit velocity was significantly greater following stimulation over the PMv during imitation compared with stimulation over the PMv during observation, regardless of action meaning. Similar effects were not observed over the PMd or vertex. In addition, stimulation over the PMv increased finger movement speed in a (non-imitative) finger–thumb opposition task. We suggest that claims regarding the role of the PMv in object-directed hand shaping may stem from the prevalence of object-directed designs in motor control research. Our results indicate that the PMv may have a broader role in ‘target-directed’ hand shaping, whereby different areas of the hand are considered targets to act upon during intransitive gesturing.

## 1. Introduction

The premotor cortex is important for action and action cognition [1]. The dorsal section (PMd) is involved in reaching and action selection [2–7]. The ventral section (PMv) is often associated with hand movements, particularly grasping and object manipulation [8–12]. The premotor cortex has also been associated with action imitation [13–15] and action recognition ([16–18], but see [19,20]). In particular, the PMv has been suggested to be part of the putative human mirror neuron system, and some claim that the PMv is the human homologue for macaque area F5 [21–23], where mirror neurons were originally observed [24]. F5 has been shown to contain neurons that code for specific hand–object interactions, rather than their single constituent movements ([25], but see [26–28]). Put simply, mirror neurons in area F5 may code hand–object oriented action, regardless of their visual or motor modality [29]. In this case, the PMv may assist in imitation by providing the scaffolding for linking observed and to-be-performed goal-directed actions [30]. There is also evidence for a role of the PMd in imitation [14], with some suggesting that it might also be involved in goal-directed action imitation [31].

The role of premotor regions in the imitation of actions that are not object- or goal-directed is still unclear. This is partly due to the fact that neuroimaging studies of action imitation are frequently focused on goal-directed or object-directed behaviour ([13], but see [32–44]). Furthermore, it is still unclear whether premotor mirror neurons can feasibly support the imitation of intransitive or novel actions. While there is some evidence for monkey mirror neuron activation in intransitive action observation [26–28], the actions used in these experiments were typically stereotyped movements (i.e. precision grasp) in the absence of an object, or non-grasps performed towards a familiar workspace. Such actions are qualitatively different to the great variety of intransitive, communicative, novel or non-functional hand shapes that humans can copy with ease. What role could the PMv and PMd play in the imitation of intransitive (meaningful) gestures or novel (meaningless) actions?

Answers to this question from neuroimaging studies have been mixed at best, with the bilateral, though more frequently left lateralized, premotor cortex associated with both meaningful and meaningless action imitation [34,35,39,42,43,45,46]. Some have suggested that the left PMv may be involved in both meaningful and meaningless action imitation [42], while others observed no activity in this region [46]. Suggestions that the PMd might be involved in action planning [13] could similarly indicate a role in both types of action imitation, but this is yet to be experimentally confirmed. Research in apraxia (a disorder of complex movement) is also worth consideration here, because it suggests that the imitation of different types of action may be processed by different areas of the brain [47–49]. However, results in apraxia are also variable. Large-scale studies of patients with apraxia comparing meaningful and meaningless action imitation indicate that damage to the left premotor cortex can be associated specifically with deficits in meaningful action imitation [50,51], or with both tasks [52].

Such variability in both the results and the experimental approaches used (i.e. neuropsychological studies; fMRI contrasts testing imitation versus rest, imitation versus observation or meaningful versus meaningless action imitation) suggests that the causal inference provided by repetitive transcranial magnetic stimulation (rTMS) might be useful to clarify the role of the left premotor cortex in imitation. Furthermore, because single participant imitation paradigms may limit our understanding of imitation as a dynamic social experience [53–55], two-person motion-tracking might help us better understand the changes in imitation behaviour that might occur following magnetic stimulation of the left premotor cortex.

We aimed to examine the role of the left premotor cortex in meaningful and meaningless action imitation, using an exploratory rTMS and motion-tracking approach that we have previously used to examine the inferior parietal lobule [56]. We chose to examine the left PMv and PMd, considering that these regions in the left hemisphere have been associated with both meaningful and meaningless action imitation [34,42,43,45,46]. We examined the effects of rTMS over these areas during both action observation and imitation, in order to better assess the specific role of these regions in imitation. If, for example, the putative mirror neuron processes performed by the PMv are essential for both recognition and imitation of known actions, we could reasonably expect rTMS over the PMv during both action observation and imitation to influence the performance of meaningful actions. We might also expect rTMS over the PMd to influence the arm movement, but not hand shaping, elements of action imitation, given the role of this area in planning reach trajectories [1]. However, we had no strong specific hypotheses, considering the large variability in results from previous experiments. Mainly, we hoped that our categorization of action into semantic, rather than goal-directed terms, along with motion-tracking the arm and hand in two interacting individuals, would develop our understanding of the role of the PMd and PMv in realistic meaningful and meaningless action imitation. We assessed imitation by examining both imitation kinematics (the movements of a participant imitator) and imitation accuracy (the correspondence between confederate actor and imitator movements).

## 2. Material and methods

### 2.1. Participants

We recruited 12 right-handed participants from the University of Nottingham and the surrounding area (mean ± s.e. age = 25.1 ± 1.7 years, four males). The experimental procedures were approved by the local ethics committee (ref: SoPEC 853); participants gave written, informed consent and the experiments were conducted in accordance with the Declaration of Helsinki (as of 2008).

### 2.2. Stimuli

**Figure 1.**
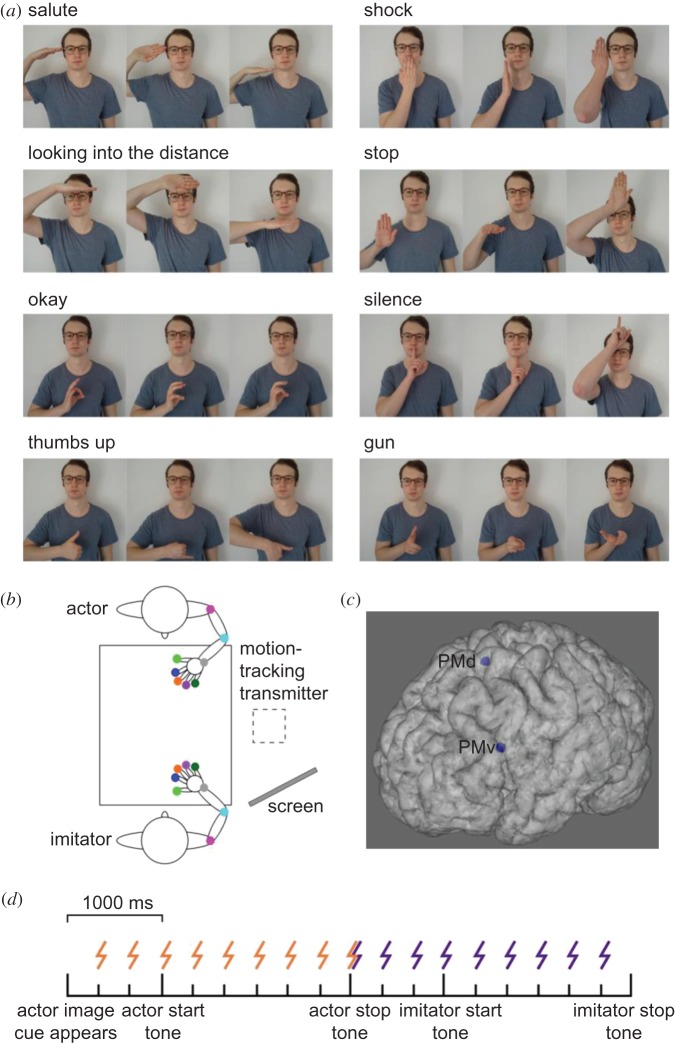
Stimuli, experimental set-up, rTMS sites and experimental timeline. (*a*) Stimuli. For each meaningful gesture, two matched meaningless gestures were created (*b*) Experimental set-up. Coloured dots indicate the location of motion trackers. The tracking box was placed next to the table, and the actor's actions were cued through images displayed on a computer screen that was not observable to the imitator. (*c*) 95% confidence ellipsoids for the rTMS target sites shown on a representative participant's brain. (*d*) Experimental timeline and stimulation onset times. Orange, observation; indigo, imitation.

A total of 24 gestures were used as stimuli. This included four meaningful hand gestures (‘salute’, ‘shock’, ‘looking into the distance’ and ‘stop’), four meaningful finger gestures (‘okay’, ‘thumbs up’, ‘silence’ and ‘gun’) and 16 matched meaningless gestures (figure 1*a*). For each meaningful gesture, two matched meaningless gestures were created. In the case of finger gestures, the matching was done by changing the fingers used to create the gesture and/or the orientation of the hand. In the case of hand gestures, matching was done by either changing the orientation or position of the hand. A well-practiced male confederate actor performed these actions for every participant.

### 2.3. Motion-tracking

Positions of the participant imitator's right arm and hand and the confederate actor's left arm and hand were recorded using a Polhemus Liberty motion tracking system (Polhemus, Inc., Colchester, VT, USA) recording 16 channels (eight per person) with 6 d.f. (*x*, *y*, *z*, azimuth, elevation and roll) at 240 Hz. Trackers were attached to the shoulder (acromial end of the clavicle), elbow (olecranon), wrist (pisiform) and the thumb and finger tips (figure 1*b*), using an adhesive medical tape or Velcro™.

### 2.4. Transcranial magnetic stimulation and electromyography

Visualization of the participant's brain was performed using T1-weighted MR images alongside the Brainsight stereotactic system (Rogue Research, Inc., Montreal, QC, Canada). To account for differences in individual anatomy, experimental stimulation locations were based on gross neuroanatomy, rather than atlas coordinates (figure 1*c*). The PMd location was defined as the anterior portion of the precentral gyrus, above the posterior limit of the superior frontal sulcus. The PMv location was defined as the anterior portion of the precentral gyrus, below the posterior limit of the inferior frontal sulcus. The vertex was used as a control site, the location of which was found using standard measures (i.e. halfway between both the two ears and the inion and nasion).

rTMS was applied using a Magstim Rapid^2^ (The Magstim Company, Cardiff, UK) with one of two 75 mm outer diameter figure-of-eight precision coils. To ensure any TMS effects were not due to motor cortical stimulation, muscle activity was recorded continuously in the right first dorsal interosseus and brachioradialis using an AD Instruments Powerlab 16/30 at 2 kHz via a Dual Bioamp/stimulator and LabChart software, with 10–500 Hz bandpass filtering.

During stimulation over the PMd, the coil was angled such that the wings did not overlap with the primary motor cortex, as measured by motor-evoked potential (MEP) presence in electromyography (EMG). In the case of MEPs being observed with rTMS applied during natural finger–thumb opposition movements (i.e. repeatedly touching the tip of the thumb to each of the fingertips), stimulation was reduced by 5% of the maximum stimulator output (MSO). If MEPs were still observed, the coil was moved medially by up to 1 cm until no MEPs could be observed.

rTMS over PMv is uncomfortable [57], so we prioritized participant comfort rather than maintaining a consistent coil orientation. During stimulation over the PMv, the coil was angled with the handle pointed towards the posterior of the skull, but manipulated to maximally reduce facial twitching and discomfort for the participant. While there is evidence to suggest that coil orientation can activate different interneuron circuits when TMS is applied over M1 (e.g. [58]), as far as we are aware the effects of coil orientation on the premotor cortex are not yet established. Furthermore, the coil was not manipulated beyond 90°. If participant discomfort was still too great, stimulation was reduced by 5% MSO.

In the imitation task, biphasic rTMS was applied over the PMv, PMd, and the vertex in a control condition for 3 s per trial at 3 Hz, and at 110% of distance adjusted resting motor threshold (RMT) [59]. The distance from M1, PMv and PMd to the outside of the skull was measured using the Brainsight neuronavigation software. RMT was obtained using the Rossini *et al*. method [60] at the start of the first session. Mean RMT was 68 ± 2.9% MSO. In each condition, stimulation intensity was limited to 80% MSO to reduce coil overheating. Stimulation was applied during either action observation or imitation, to examine the role of each area in these separate components of the imitation task. Vertex stimulation intensity was taken as the mean of PMd and PMv. Mean ± s.e. stimulation intensity in each condition was as follows: PMv = 65 ± 2.6%, PMd = 74 ± 2.4%, vertex = 72 ± 2.1% MSO.

### 2.5. Design and procedure

The experiment was controlled and data were acquired using custom software written in Labview (National Instruments). We used LabMan (custom in-house software) to document experiments, and the HandLabToolbox (https://github.com/TheHandLab/HandLabToolBox) and Matlab 2016b (Mathworks, Inc.) to analyse data.

During the imitation task, participants sat opposite the confederate at a rectangular plastic table, approximately 76 cm away from each other (figure 1*b*). A start point was located 20 cm away from each individual's abdomen using Blu Tack^®^. To inform the confederate actor of the action they needed to perform, a computer screen was placed behind the imitator. This was unobservable by the participant imitator.

Participants took part in three sessions, counterbalanced across participants and separated by at least 24 h (mean ± s.d. = 4.58 ± 3.90 days between the first and last session). Within each session, a single brain region was stimulated, and participants took part in both meaningful and meaningless action imitation tasks. Since there is evidence to suggest that performing novel and known actions in a sequence could recruit a single processing route, while presenting them separately recruits separate routes ([61], but see [62], and [63]), meaningful and meaningless action trials were split into separate blocks. Task order was counterbalanced across stimulation sites.

At the start of each of the three sessions, we requested participants to perform a finger–thumb opposition task while their finger and arm movements were tracked and EMG was recorded over the right first dorsal interosseus and brachioradialis. The finger–thumb opposition task consisted of repetitively touching the tip of the thumb to each of the fingertips back-and-forth across the hand (index finger, middle finger, ring finger, little finger, ring finger, middle finger, etc.). Participants were requested to perform this task as quickly and as accurately as possible for 15 s. During the middle section of this task, rTMS was applied at 3 Hz (15 pulses, from 5.00 to 9.67 s) at the chosen intensity for that session's brain region. The purpose of this motor task was twofold. Firstly, we wanted a measure of performance in a control motor task to examine the role of the stimulated regions in more general fine-motor control. Additionally, by observing the EMG data online during the finger–thumb opposition task, we could make changes to the angle of the coil or intensity of stimulation in the case of unwanted M1 stimulation (i.e. TMS-evoked MEPs).

During the imitation task, both confederate and participant imitator began with their thumb and forefinger gripping their start points. In both meaningful and meaningless imitation tasks, action images appearing on a computer screen in a random order informed the confederate of which action to perform. The screen was unobservable by the imitator. A tone 1000 ms after image presentation signalled the actor to begin the action, which they performed and maintained until a second, lower frequency tone was played 2000 ms later (figure 1*d*). They then returned their hand to the start point. 1000 ms after the signal for the actor to return their hand, a tone played to signal the imitator to imitate the action which they performed and maintained until a second, lower frequency tone was played 2000 ms later. They then returned their hand to the start point. A total of 64 trials were presented in this way, and the imitator was provided with a break at the halfway point. rTMS occurred during action observation or imitation (figure 1*d*). Stimulation during observation started 333 ms after the point at which the new image appeared on the screen, meaning that stimulation was performed prior to action observation, and during the whole observation period. Stimulation during imitation started 1000 ms before the imitator was cued to begin their action, at the same relative point in time at which observation stimulation finished, and ending 333 ms before the participant was cued to finish maintaining the gesture and return their hand. This ensured that the number of pulses was matched for both conditions. There was a 10 s gap between the end of one and the start of the following train of stimulation, with trial timings matched to this criterion.

Following the completion of all TMS sessions, participants were presented with a questionnaire featuring the meaningful and meaningless images in a pseudorandom order, with the aim to exclude participants if they were less than 60% consistent with our own categorization of the actions. No participants were excluded on this criterion. Mean ± s.e. rating agreement was 75.0 ± 6.15% for meaningful actions and 86.5 ± 2.86% for meaningless actions.

### 2.6. Data analysis

For the finger–thumb opposition task, an automated analysis routine processed the position data from each trial of each participant and rejected artefacts (e.g. trials with missing samples or spikes resulting from electromagnetic interference). Single timepoint spikes (greater than 3 s.d. from the mean), in each trial's double-differentiated time-series were deemed electromagnetic artefacts and removed by interpolation across three adjacent samples either side. The data were filtered using a bidirectional low-pass fourth-order Butterworth filter, with a cut-off frequency of 12 Hz.

We took three measures of task performance in the finger–thumb opposition task: the average three-dimensional (3D) velocity of the fingers throughout the task, the 3D velocity of the thumb throughout the task, the inter-touch interval (between each digit and thumb, across the required movement pattern) and the inter-touch interval s.d. Values were calculated for each of the brain stimulation sites for 5 s pre-stimulation, 5 s stimulation and 5 s post-stimulation. The data were analysed using a two-way repeated-measures ANOVA using stimulation site and time as the within-subjects factors.

An automated script was also used for pre-processing and extraction of variables in the imitation task. Artefacts in the data were removed using the double-differentiation detection and interpolation approach as in the finger–thumb opposition analysis above. Since this approach did not result in complete removal of all artefacts, we then used a locally weighted scatter plot smoothing method. This was done with the Matlab ‘smooth’ function, using a weighted linear least squares and a first-degree polynomial model over a moving window of five samples to identify outliers.

Unfortunately, a large number of TMS-related artefacts remained in the position data for the imitation experiment. Since these artefacts were time-locked to the stimulation onsets for each participant, we removed the data including and surrounding these timepoints for every trial for each participant, based on their individual artefact onsets. Removed data points were interpolated using spline interpolation, and then every trial was inspected to confirm complete removal of artefacts and normal trajectory shape. This pre-processing step examined the entire time-series (effectively 1800 ms, the maximum accepted movement time), including data prior to the movement onset and after the movement end (which were not used in final analysis). The largest continuous series of interpolated points in any instance for the actor data was 53 samples (221 ms). The mean ± s.e. sum of the interpolated samples in each trial for the actor data was 165 ± 9 (688 ± 37.5 ms) participant-wise, while the mean ± s.e. length of any single interpolated period for the actor data was 28 ± 1 (117 ± 4.17 ms). The largest continuous series of interpolated points in any instance for the imitator data was 33 samples (138 ms). The mean ± s.e. sum of the interpolated samples in each trial for the imitator data was 110 ± 5 (458 ± 20.8 ms) participant-wise, while the mean ± s.e. length of any single interpolated period for the imitator data was 19 ± 1 (79.2 ± 4.17 ms).

The data were then filtered with a bidirectional low-pass fourth-order Butterworth filter (cut-off frequency 12 Hz). Any trials in which the participant started moving before the starting tone, or failed to finish moving within 200 ms of the return tone, were excluded. Trials in which any of the trackers had an unusually high velocity (greater than 250 cm s) were also excluded. Following the above exclusions, a total of 83.4% of trials were maintained for statistical analysis.

We examined two elements of participant behaviour: imitation accuracy and imitation kinematics. To test imitation accuracy, we compared the actor and the imitator 3D velocity for each of the trackers over their primary movement (movement onset to gesture completion, based on a 12 cm s threshold). To do this, we ran a correlation between resampled actor and imitator velocity curves for each trial for each tracker. Velocity curves were resampled to 120 samples and normalized in amplitude (by dividing each value by the maximum velocity). To allow parametric analysis, the resulting *r*-values were converted to *Z*-values using the Fisher transformation (*Z* = 0.5 * ln(1 + *r*/1 − *r*), where ln is the natural logarithm). The means of the *Z*-values for each condition were analysed using a four-way (stimulation site, stimulation time, meaning, effector) repeated-measures ANOVA.

To reduce our likelihood of reporting false positives, we divided our *α*-value cut-off for assessing statistical significance by 8 (the number of trackers). Therefore, in the ANOVAs of *Z*-values, the value of *α* used to determine a significant result was reduced from 0.05 to 0.0063.

To assess imitator kinematics for time-dependent significant differences in the stimulation-related main effects or interactions, *t*-statistic plots were created for each instance. We took this time-series-driven approach in order to inform us of possible differences in peak kinematic values in separate trackers, without the inflated type 1 error that would occur were we to examine multiple kinematic parameters in multiple trackers. This approach has been used to assess changes in imitation kinematics following rTMS [56].

To create these *t*-statistic plots, the imitator's 3D velocity over their primary movement was resampled to 120 samples. In cases where the *t*-value was at a significant level for any sequence of samples in the time-series, we performed permutation testing. Permutation testing was performed over 10 000 iterations to create a custom empirical null distribution of the length of samples, taken from randomly selected trials, with significant *t*-statistics, which was then used to decide whether an observed sequence in the original dataset was significantly long. This is similar to the use of cluster-based statistics in fMRI, where a fixed, arbitrary threshold is used for creating clusters, then a second threshold is calculated for determining how large a cluster needs to be before it is statistically significant. On each iteration, the condition labels for each participant's data were pseudorandomized, and the original analyses were then repeated exactly, in order to obtain *t*-statistics, and sequences of significant *t*-statistics for the difference between conditions, under the null hypothesis. From this, we were able to assign a *p*-value to our actual results by seeing what proportion of the tail of the distribution was greater (or lesser) than or equal to the actual result. We examined the minimum length of sequences of continuous values in which |*t*| > 2.201 (i.e. statistically significant at a sample-wise level, *p* < .05), and also the *p*-values associated with the sequences of timepoints in our recorded data where |*t*| > 2.201.

In cases where the *t*-value was at a significant level for any period of the time-series, we confirmed whether these sequences overlapped with any peak kinematic parameters (peak acceleration, PA; velocity, PV; or deceleration, PD), on which we ran two-way paired *t*-tests in the original (non-resampled) data, Bonferroni-correcting where necessary. This allowed us to ascertain whether the differences were derived from the PMv–PMd comparison, or if there was further information to be gained from the vertex control site. To account for potential confederate bias, we also tested whether similar effects were observed in *t*-statistic plots for the actor. Actor *t*-statistic plots were created from the actor primary movement velocity profiles in the same way as the imitator *t*-statistic plots. For all *post hoc* paired *t*-tests, Hedges' *g*_rm_ was chosen to report effect sizes in repeated-measures comparisons [64].

## 3. Results

### 3.1. Finger–thumb opposition task

A two-way repeated-measures ANOVA revealed that for finger velocity throughout the task, there was a significant effect of stimulation site (table 1). *Post hoc* paired *t*-tests using a Bonferroni-corrected *α* of 0.025 revealed that finger velocity was significantly greater overall for the PMd condition compared with the vertex (*t*_11_ = 5.52, *p* < 0.001, *g*_rm_ = 0.285). There was no significant difference between PMv and vertex (*t*_11_ = 2.26, *p* = 0.045, *g*_rm_ = 0.214), or between PMv and PMd (*t*_11_ = −1.07, *p* = 0.307, *g*_rm_ = 0.0938). While the ANOVA did not reveal a significant effect of time (pre-stimulation, stimulation, post-stimulation), there was a statistically significant site * time interaction.

**Table 1.** Mean values and ANOVA results for all variables assessed in the finger–thumb opposition task; statistically significant effects in bold.

	mean (±s.e.) value																	
	stimulation site			time			main effect of stimulation site				main effect of time				site * time interaction			
	PMv	PMd	vertex	pre	stimulation	post	*F*	d.f.	*P*	***η***^2^	*F*	d.f.	*p*	***η***^2^	*F*	d.f.	*p*	***η***^2^
finger velocity (cm s)	5.67 (0.683)	5.94 (0.774)	5.10 (0.726)	5.81 (0.666)	5.89 (0.811)	5.01 (0.862)	7.31	2, 22	**0.004**	0.399	1.57	2, 22	0.231	0.125	3.02	4, 44	**0.028**	0.215
thumb velocity (cm s)	5.54 (0.486)	6.24 (0.645)	5.69 (0.662)	6.02 (0.667)	5.63 (0.538)	5.82 (0.656)	3.71	1.16, 12.7	0.072	0.252	0.520	2, 22	0.602	0.045	1.23	1.99, 21.9	0.313	0.100
inter-touch interval (ms)	348 (20.1)	359 (26.2)	354 (20.3)	347 (19.2)	346 (20.1)	369 (23.9)	0.301	1.22, 13.4	0.637	0.027	7.53	2, 22	**0.003**	0.406	1.38	4, 44	0.257	0.111
inter-touch interval s.d. (ms)	74.2 (13.4)	77.4 (12.1)	84.0 (12.2)	67.6 (10.2)	68.5 (9.54)	99.5 (16.1)	0.352	2, 22	0.707	0.031	5.57	1.36, 15.0	**0.024**	0.336	0.591	2.01, 22.2	0.563	0.051

**Figure 2.**
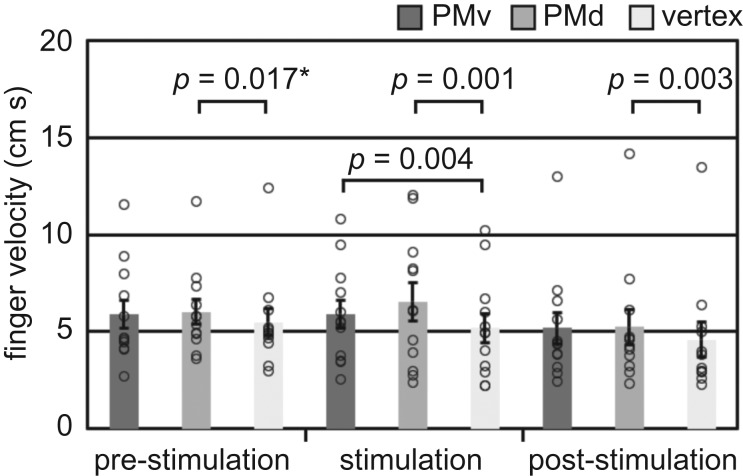
Finger velocity during the finger–thumb opposition task. Error bars indicate standard error, while single points show individual participant values. Comparisons indicated with an asterisk were not significant at the Bonferroni-corrected *α* threshold of 0.006.

*Post hoc* paired *t*-tests were used to further examine this interaction, using a Bonferroni-corrected *α* of 0.006 (figure 2). There was a significant increase in finger velocity for PMv compared with the vertex during stimulation (*t*_11_ = 3.59, *p* = 0.004, *g*_rm_ = 0.256), but not prior to stimulation (*t*_11_ = 1.15, *p* = 0.274, *g*_rm_ = 0.151) or post-stimulation (*t*_11_ = 2.00, *p* = 0.071, *g*_rm_ = 0.182). There was no significant difference in finger velocity for PMv compared with PMd pre-stimulation (*t*_11_ = 0.391, *p* = 0.704, *g*_rm_ = 0.0477), during stimulation (*t*_11_ = 2.00, *p* = 0.071, *g*_rm_ = 0.125) or post-stimulation (*t*_11_ = 0.250, *p* = 0.807, *g*_rm_ = 0.0158). There was, however, a significantly greater finger velocity for the PMd compared with the vertex during stimulation (*t*_11_ = 4.41, *p* = 0.001, *g*_rm_ = 0.285) and post-stimulation (*t*_11_ = 3.86, *p* = 0.003, *g*_rm_ = 0.191), but not at the corrected *α* threshold pre-stimulation (*t*_11_ = 2.81, *p* = 0.017, *g*_rm_ = 0.202). Similar effects were not observed in the ANOVA of thumb velocity (table 1). These results suggested that, while finger velocity was higher in the PMd condition compared with the vertex in general, a stimulation-specific effect was observed for the PMv. When stimulation was applied over the PMv, participants increased the speed with which they moved their fingers.

Examining the inter-touch interval (ITI) showed that there was a significant main effect of time (table 1). *Post hoc* paired *t*-tests using a Bonferroni-corrected *α* of 0.025 revealed that the ITI was significantly greater post-stimulation (369 ± 23.9 ms) compared with both pre-stimulation (347 ± 19.2 ms, *t*_11_ = 2.78, *p* = 0.018, *g*_rm_ = 0.229) and stimulation (346 ± 20.1 ms, *t*_11_ = 3.39, *p* = 0.006, *g*_rm_ = 0.237). There was no significant difference between pre-stimulation and stimulation (*t*_11_ = 0.166, *p* = 0.871, *g*_rm_ = 0.0116). Effects in the same direction were observed in the ANOVA of ITI s.d. (table 1). The ITI s.d. was significantly greater post-stimulation (99.5 ± 16.1 ms) compared with during stimulation (68.5 ± 9.54 ms, *t*_11_ = 2.68, *p* = 0.022, *g*_rm_ = 0.552). A similar effect that did not meet the Bonferroni-corrected *α* threshold was observed between post-stimulation and pre-stimulation (67.6 ± 10.2 ms, *t*_11_ = 2.38, *p* = 0.036, *g*_rm_ = 0.599). As was observed for ITI, there was no significant difference between pre-stimulation and stimulation (*t*_11_ = −0.144, *p* = 0.888, *g*_rm_ = 0.0252). These results suggest that, regardless of stimulation site, participants increased both the time between touches and the variability of that time following rTMS, possibly due to a distracting effect of the stimulation.

### 3.2. Imitation task

#### 3.2.1. Actor–imitator correspondence

There were no statistically significant effects of stimulation site or stimulation time in the correlation analysis (electronic supplementary material, table S1). Meaningless actions were significantly better correlated than meaningful actions in the shoulder (mean ± s.e. *z*-value = 0.580 ± 0.0457 versus 0.422 ± 0.0427, *F*_1,11_ = 22.9, *p* = 0.001, *η*^2^ = 0.675), indicating that participants matched the actor's shoulder movements better when the action was meaningless. An effect in the same direction that was significant using an *α* value of 0.05, but not the corrected value of 0.0063, was observed in the elbow (*p* = 0.030).

Meaningful actions were significantly better correlated than meaningless actions in the middle (1.68 ± 0.0394 versus 1.56 ± 0.0443, *F*_1,11_ = 17.9, *p* = 0.001, *η*^2^ = 0.619), ring (1.73 ± 0.0312 versus 1.63 ± 0.0372, *F*_1,11_ = 16.8, *p* = 0.002, *η*^2^ = 0.605) and little fingers (1.76 ± 0.0353 versus 1.68 ± 0.0393, *F*_1,11_ = 12.9, *p* = 0.004, *η*^2^ = 0.539), indicating that participants matched the actor's finger movements better when the action was meaningful. Effects in the same direction that were significant at *α* 0.05, but not 0.0063, were observed in the wrist (*p* = 0.007), thumb (*p* = 0.021) and index finger (*p* = 0.012) trackers.

Hand gestures were significantly better correlated than finger gestures in the shoulder (0.683 ± 0.0499 versus 0.319 ± 0.0378, *F*_1,11_ = 119, *p* < 0.001, *η*^2^ = 0.915), elbow (1.19 ± 0.0500 versus 0.804 ± 0.0630, *F*_1,11_ = 109, *p* < 0.001, *η*^2^ = 0.908) and index finger (1.70 ± 0.0434 versus 1.46 ± 0.0352, *F*_1,11_ = 37.5, *p* < 0.001, *η*^2^ = 0.773). An effect in the same direction that was significant at *α* 0.05, but not 0.0063, was observed in the wrist (*p* = 0.047), thumb (*p* = 0.007), ring (*p* = 0.017) and little finger (*p* = 0.020) trackers. This indicates that participants matched the actor's arm and hand movements better when they had to copy a hand gesture. There were no statistically significant interactions in the correlation analysis (electronic supplementary material, tables S2 and S3).

#### 3.2.2. *T*-statistic plots and permutation testing

**Figure 3.**
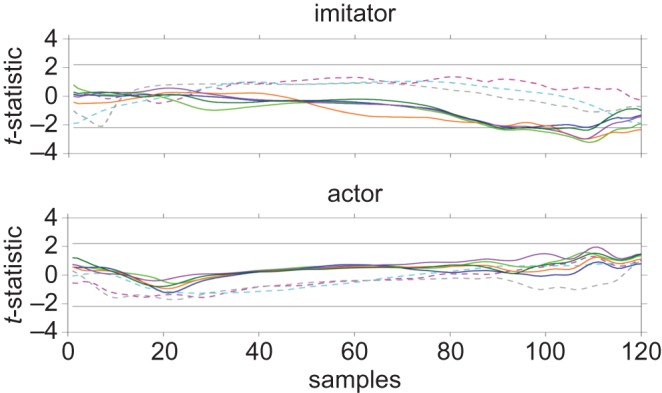
*t*-statistic plots for main effect of stimulation site (PMv versus PMd) in all trackers. Horizontal lines indicate positive and negative critical *t*-values; Dashed magenta, shoulder; dashed cyan, elbow; dashed grey, wrist; light green, thumb; blue, index finger; orange, middle finger; purple, ring finger; dark green, little finger.

When comparing the resampled velocity curves between PMv and PMd conditions (figure 3), there were significantly long sequences for the thumb between 89 and 117 samples (*p* = 0.026), middle finger between 102 and 120 samples (*p* = 0.048), and little finger between 91 and 110 samples (*p* = 0.041), such that the velocity with TMS over the PMv was greater than with TMS over the PMd. Sequences between 93 and 95 samples (*p* = 0.204) in the index finger and between 99 and 112 samples (*p* = 0.078) in the ring finger were not statistically significant. These periods overlapped with the deceleration phase on resampled velocity curves (electronic supplementary material, figure S1), and no similar effect was observed when we performed the same *t*-statistic plotting on the actor data (figure 3), indicating that this effect was confined to the participant imitator and that the actor was not biased in the same direction.

Since these periods overlapped with the deceleration phase, we decided to examine the original mean digit PD using Bonferroni-corrected *t*-tests with an *α* cut-off of 0.025, in order to confirm if these effects could be observed in the original (non-resampled) data. However, there was no significant difference in mean digit PD between PMv and PMd (mean ± s.e. = −582 ± 38.0 versus −578 ± 30.4 cm s, *t*_11_ = −0.173, *p* = 0.044, *g*_rm_ = 0.0228), between PMv and vertex (−564 ± 22.6 cm s, *t*_11_ = −0.668, *p* = 0.518, *g*_rm_ = 0.135) or between PMd and vertex (*t*_11_ = −0.617, *p* = 0.550, *g*_rm_ = 0.137).

**Figure 4.**
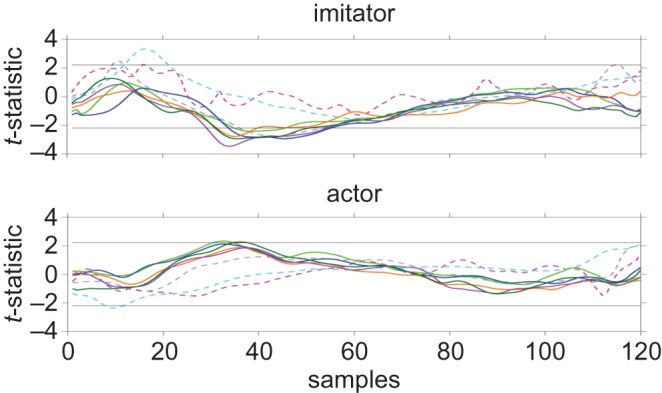
*t*-statistic plots for site * time interaction in all trackers. Horizontal lines indicate positive and negative critical *t*-values; Dashed magenta, shoulder; dashed cyan, elbow; dashed grey, wrist; light green, thumb; blue, index finger; orange, middle finger; purple, ring finger; dark green, little finger.

In the site * time interaction (figure 4), there were significantly long sequences present in the index finger from samples 34–55 (*p* = 0.044) and ring finger from samples 31–54 (*p* = 0.036). These sequences overlapped with the period covering PV in resampled velocity curves (electronic supplementary material, figure S2). Similar effects were not observed in the actor data (figure 4). While the sequences in the imitator thumb (*p* = 0.109), middle finger (*p* = 0.126) and index finger (*p* = 0.054) were not significantly long, the consistent direction of effects observed prompted us to examine the original mean digit PV using two-tailed *t*-tests, in order to better understand the effects of stimulation over different brain regions at different times.

**Figure 5.**
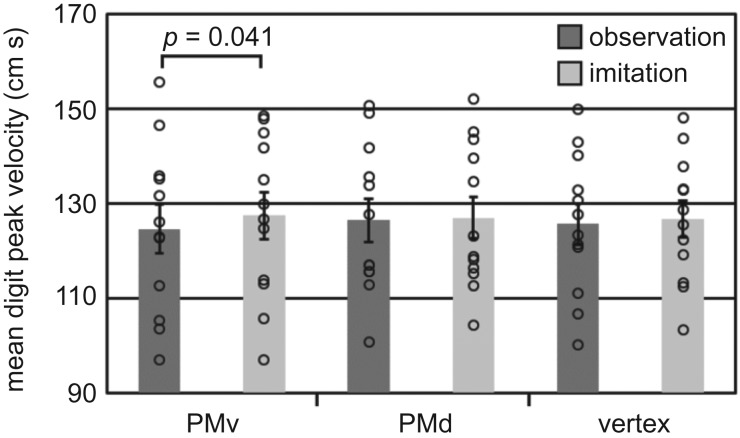
Imitator mean digit peak velocity. Error bars indicate standard error, while single points show individual participant values.

Importantly, this revealed that mean digit PV was significantly greater (figure 5) following stimulation over the PMv during imitation compared with stimulation over the PMv during observation (*t*_11_ = −2.31, *p* = 0.041, *g*_rm_ = 0.149). There was no significant difference between stimulation over the PMd during observation and imitation (*t*_11_ = −0.622, *p* = 0.547, *g*_rm_ = 0.0314), or between stimulation over the vertex during observation and imitation (*t*_11_ = −0.961, *p* = 0.357, *g*_rm_ = 0.0633).

Finally, there was a significantly long sequence observed for the site * effector interaction (electronic supplementary material, figure S3B) in the elbow from samples 31–69 (*p* = 0.022) overlapping PA, PV and PD, though no significant differences were found in elbow peak values when we examined these using Bonferroni-corrected *t*-tests (electronic supplementary material, table S4). There were no other significantly long sequences observed in the *t*-statistic plots (electronic supplementary material, figure S3).

## 4. Discussion

In this experiment, we examined the role of the left dorsal and ventral premotor cortices in meaningful and meaningless action imitation. We were interested to see the degree to which these areas might distinguish between meaningful and meaningless actions for the purpose of imitation. We found that stimulating the PMv increased the movement speed of the digits, both in our control finger–thumb opposition and our imitation task (regardless of action meaning). We also revealed novel effects of action meaning and effector on imitation accuracy.

### 4.1. Role of the ventral premotor cortex in hand shaping

In our imitation task, we found that mean digit peak velocity was significantly greater following stimulation over the PMv during imitation compared with stimulation over the PMv during observation. There was no significant difference between stimulation over the PMd during observation and imitation, or between stimulation over the vertex during observation and imitation. Additionally, in our control fine motor task, the finger–thumb opposition task, we also found that stimulating the PMv speeded finger movements. This effect was specific to the PMv because, while similar effects were observed for the PMd, the PMd condition was different from the vertex even pre- and post-stimulation, suggesting that the PMd effects were not stimulation-specific. These results are broadly supportive of a general role for the PMv in fine motor control of the digits, which is not specific to imitation.

The PMv is often associated with hand shaping during grasping [8–12]. One possibility that arises from our results is that the hand-shaping element is not object-specific, and can be associated with other hand-shaping movements that are directed towards more abstract goal-states (like gesture formation). Hoshi & Tanji [65] claimed that during ‘direct sensorimotor processing, the PMv receives information on a motor target and sends outputs to achieve an action that directly matches the information’ (p. 240), while Vingerhoets & Clauwaert [66] suggested that the PMv has a role in ‘matching hand posture configurations in accordance to visual demands' (p. 3437). However, neither of these descriptions are necessarily reliant on a target object. We posit that during both meaningful and meaningless gesture formation, the movement of the digits towards other digits or the palm of the (same) hand is enough to constitute a ‘target-directed’ hand shaping action. In this explanation, the other digits or parts of the hand constitute the object/target. As in object-directed action [10], the PMv would process target-relevant properties for hand shape and possibly transmit this information to M1 in a muscle-specific manner. The manipulation of this process following rTMS during action performance could therefore result in increased muscle recruitment, leading to the increase in finger velocity observed here. It is worth noting the failure of PMv stimulation to influence imitation accuracy, despite influencing digit velocity. As we have observed previously [56], it is possible that rTMS is not powerful enough to meaningfully influence imitation accuracy, hence effects are observed solely in relatively subtle modulations of kinematic landmarks.

We are unaware of any other studies that have causally linked (i.e. using neurostimulation) the PMv to gestural kinematics using motion-tracking (but see [67] for a role of the PMv in compensating grip aperture perturbation). However, our claim is broadly in keeping with some previous discussion [45]. It seems possible then that popular consideration of the PMv in terms of object-directed hand shaping is purely a result of the object-directed experimental paradigms commonly used to examine this region. The necessity of hand shaping for all types of hand gesture imitation might provide an explanation for why the PMv is frequently reported in neuroimaging studies of imitation (see the section ‘Introduction’). A more targeted rTMS study, aimed at confirming the role of the left PMv in hand shaping for different types of hand actions outside of an imitative context, would be useful in the future.

A general role for the PMv in hand shaping during action is not the only feasible explanation for our results, however. It is also not possible to completely rule out a general influence of TMS, because one problem with stimulating the PMv is that it is often uncomfortable [57], though our decision to base stimulation levels on a distance-adjusted RMT meant that stimulation over the PMv was 9% MSO lower than over the PMd. As Meteyard & Holmes [57] show that the annoyance level of TMS stimulation is linearly related to MSO, the difference in MSO between the PMv and PMd (and vertex) could feasibly offset any differences in discomfort.

### 4.2. Absence of dorsal premotor cortex effects in imitation

We did not observe any significant main effects or interactions associated with PMd stimulation during imitation and, as mentioned above, differences between PMd and vertex in the finger–thumb opposition task did not appear to be stimulation-specific. The absence of an imitation-related effect was surprising, considering previous findings associating this area with intransitive action performance [68,69]. Furthermore, there is some evidence to suggest that both the PMd and PMv could play a role in encoding hand-shaping kinematics [70], and rTMS over the left PMd has been found to induce inhibition in the hand area of M1 [71].

Importantly, the more frequent discussion of the PMd in terms of reaching [1] may not be fully applicable to the gestural actions which we were using as stimuli. In particular, there are likely to be different processing routes underlying point-to-point functional actions like reaching, compared with the less linear trajectories used for abstract action, like gesture [72]. It is still unclear, then, what PMd activity in previous studies of imitation could represent. It may be that this region is more likely to be involved in the encoding of external (i.e. not part of the body) targets [31].

### 4.3. Effector specificity and gesture meaning

Testing actor–imitator correlation revealed some interesting effects of both meaning and effector, providing new contributions to our understanding of action imitation. Meaningless actions were better correlated than meaningful actions in the shoulder and elbow, replicating similar results that were observed in a previous experiment [56]. By contrast, meaningful actions were better correlated than meaningless actions in the wrist and fingers.

These results seem to allow one to infer distinctions between proximal (shoulder, elbow) and distal (wrist, digit) recruitment dependent on action meaning. In the case of meaningless action imitation, where adherence to observed kinematics may be more useful [73,74], it is likely that the shoulder and elbow are easier to imitate than the fingers because they have fewer degrees of freedom. Furthermore, because meaningful actions are already present in the motor repertoire, the proximal effectors may be less accurately imitated in order to perform the action in a preferred fashion. Alternatively, the higher degrees of freedom in the wrist and digits make matching much more difficult, hence imitation is less accurate for meaningless actions.

Hand gestures were better correlated than finger gestures in the shoulder, elbow, wrist, thumb, index finger, ring finger and little finger. These effects probably reflect the fact that hand gestures were computationally less complex. The hand moves as one single effector to a single point in the body, at a certain orientation, while finger gestures require accurately distinguishing the position and orientation of each of the digits.

### 4.4. Conclusion

Our experiment has revealed new information about the role of the left PMv in action imitation, and the general approach taken towards imitating meaningful and meaningless hand and finger gestures. The results indicate that the left PMv may have a general role in hand shaping for gesture, expanding on results from primarily object-directed studies of motor control. We suggest that in the absence of an object, as in the case of intransitive gesturing, the hand itself can act as a target for the digits to act upon. This process might recruit the PMv in a similar way to object-directed hand shaping. In addition, we observed that hand gestures are better imitated than finger gestures, probably reflecting the relative simplicity of hand actions. Meaningful actions are better imitated in distal effectors (hand, fingers), while meaningless actions are better imitated in proximal effectors (shoulder, elbow), possibly reflecting the degree to which meaningful and meaningless actions rely on close matching of the observed action.

## Supplementary Material

Supplementary Material

## Data accessibility

Raw data are available from the Open Science Framework (doi:10.17605/OSF.IO/F4ETU).
