# Correction to: ‘The left ventral premotor cortex is involved in hand shaping for intransitive gestures: evidence from a two-person imitation experiment’

**DOI:** 10.1098/rsos.181855

**Published:** 2018-11-28

**Authors:** Arran T. Reader, Nicholas P. Holmes

*R. Soc. open sci.* **5**, 181356. (Published 10 October 2018). (doi:10.1098/rsos.181356)

This correction refers to errors in the text on page 10, where ‘cm s’ has been used in place of ‘cm s^2^’, and a standard error value and *p*-value are incorrectly reported. The corrected text is shown below, and does not alter the conclusions of the article.

## 

### 3.2.2. T-statistic plots and permutation testing

However, there was no significant difference in mean digit PD between PMv and PMd (mean ± s.e. = −582 ± 38.0 versus −578 ± 30.4 cm s^2^, *t*_11_ = −0.173, *p* = 0.866, *g*_rm_ = 0.0228), between PMv and vertex (−564 ± 25.6 cm s^2^, *t*_11_ = −0.668, *p* = 0.518, *g*_rm_ = 0.135) or between PMd and vertex (*t*_11_ = −0.617, *p* = 0.550, *g*_rm_ = 0.137).

