# Supplementary material for: The left ventral premotor cortex is involved in hand shaping for intransitive gestures: evidence from a two-person imitation experiment
Source: R Soc Open Sci. 2018 Oct 10;5(10):181356. doi: 10.1098/rsos.181356 (PMC6227992; doi:10.1098/rsos.181356)
Supplement: Supplementary Material [file rsos181356supp1.pdf]

The left ventral premotor cortex is involved in hand shaping for intransitive gestures: evidence from a two-person imitation experiment

Arran T. Reader & Nicholas P. Holmes

Supplementary tables

Table 1: Mean values and main effects for actor-imitator correlation

Significant p-values (<.0063) are in bold; SH: shoulder, EL: elbow, WR: wrist, TH: thumb, IN: index finger, MI: middle finger, RI: ring finger, LI: little finger.

Tracker	Mean(±SE) Z-value									Main effect											
	Site			Time		Meaning		Effector		Site			Time			Meaning			Effector		
	PMv	PMd	Vertex	Observation	Imitation	Meaningful	Meaningless	Hand	Finger	F (2, 22)	p	η^2	F (1, 11)	p	η^2	F (1, 11)	p	η^2	F (1, 11)	p	η^2
SH	0.515 (0.0468)	0.443 (0.0573)	0.545 (0.0365)	0.503 (0.0417)	0.499 (0.0416)	0.422 (0.0427)	0.580 (0.0457)	0.683 (0.0499)	0.319 (0.0378)	3.18	.061	.224	0.065	.803	.006	22.9	**.001**	.675	119	**<.001**	.915
EL	1.01 (0.0671)	0.988 (0.0681)	0.944 (0.0590)	0.984 (0.0550)	1.01 (0.0534)	0.932 (0.0711)	1.06 (0.0460)	1.19 (0.0500)	0.804 (0.0630)	0.056	.946	.005	4.16	.066	.275	6.19	.030	.360	109	**<.001**	.908
WR	1.94 (0.0708)	1.92 (0.0675)	2.02 (0.0488)	1.95 (0.0617)	1.98 (0.0543)	2.02 (0.0666)	1.90 (0.0528)	2.00 (0.0708)	1.92 (0.0483)	2.31	.123	.174	4.80	.051	.304	10.8	.007	.496	5.02	.047	.313
TH	1.64 (0.0584)	1.63 (0.0516)	1.64 (0.0376)	1.62 (0.0480)	1.64 (0.0455)	1.67 (0.0478)	1.60 (0.0470)	1.69 (0.0548)	1.58 (0.0421)	0.020	.980	.002	1.52	.244	.121	7.18	.021	.395	10.9	.007	.497
IN	1.58 (0.0481)	1.57 (0.0450)	1.59 (0.0297)	1.57 (0.0353)	1.59 (0.0350)	1.62 (0.0369)	1.54 (0.0372)	1.70 (0.0434)	1.46 (0.0352)	0.061	.941	.005	1.01	.336	.084	9.02	.012	.451	37.5	**<.001**	.773
MI	1.63 (0.0507)	1.60 (0.0455)	1.64 (0.0399)	1.62 (0.0418)	1.63 (0.0383)	1.68 (0.0394)	1.56 (0.0443)	1.66 (0.0585)	1.59 (0.0296)	0.701	.507	.060	1.57	.237	.125	17.9	**.001**	.619	2.31	.156	.174
RI	1.67 (0.0445)	1.68 (0.0444)	1.69 (0.0291)	1.68 (0.0352)	1.68 (0.0305)	1.73 (0.0312)	1.63 (0.0372)	1.73 (0.0464)	1.63 (0.0261)	0.120	.888	.011	0.454	.514	.040	16.8	**.002**	.605	7.98	.017	.420
LI	1.71 (0.0471)	1.71 (0.0467)	1.73 (0.0315)	1.72 (0.0397)	1.72 (0.0324)	1.76 (0.0353)	1.68 (0.0393)	1.77 (0.0501)	1.67 (0.0256)	0.158	.855	.014	0.044	.838	.004	12.9	**.004**	.539	7.32	.020	.400

Table 2: Two-way interactions for actor-imitator correlation

SH: shoulder, EL: elbow, WR: wrist, TH: thumb, IN: index finger, MI: middle finger, RI: ring finger, LI: little finger.

Tracker	Site*time			Site*meaning			Site*effector				Time*meaning			Time*effector			Meaning*effector		
	F (2, 22)	p	η^2	F (2, 22)	p	η^2	F (2, 22)	df	p	η^2	F (1, 11)	p	η^2	F (1, 11)	p	η^2	F (1, 11)	p	η^2
SH	0.234	.793	.021	0.410	.668	.036	1.27	2, 22	.302	.103	0.100	.757	.009	1.41	.260	.114	7.52	.019	.406
EL	1.16	.334	.095	0.454	.61	.040	2.55	2, 22	.101	.188	0.242	.633	.022	2.44	.147	.181	0.050	.828	.004
WR	0.270	.765	.024	0.488	.620	.042	1.02	2, 22	.376	.085	2.20	.101	.225	0.267	.615	.024	0.003	.955	<.001
TH	0.827	.451	.070	0.501	.613	.044	0.078	1.30, 14.3	.846	.007	0.587	.260	.051	0.239	.635	.021	5.08	.046	.316
IN	0.852	.440	.072	1.23	.312	.101	0.047	2, 22	.892	.010	0.033	.858	.003	0.009	.924	.001	4.73	.052	.301
MI	0.872	.432	.073	1.67	.211	.132	0.166	2, 22	.848	.015	2.37	.152	.177	0.029	.867	.003	7.22	.021	.396
RI	1.35	.279	.110	3.16	.062	.223	0.183	2, 22	.834	.016	1.50	.246	.120	0.001	.981	<.001	4.93	.048	.309
LI	2.74	.086	.200	3.06	.067	.218	0.383	2, 22	.687	.034	4.19	.065	.276	0.729	.411	.062	7.16	.022	.394

Table 3: Three- and four-way interactions for actor-imitator correlation

SH: shoulder, EL: elbow, WR: wrist, TH: thumb, IN: index finger, MI: middle finger, RI: ring finger, LI: little finger.

Tracker	Site*time* meaning				Site*time* effector			Site*meaning* effector			Time*meaning* effector			Site*time* meaning*effector		
	F	df	p	η^2	F (2, 22)	p	η^2	F (2, 22)	p	η^2	F (1, 11)	p	η^2	F (2, 22)	p	η^2
SH	0.795	1.30, 14.3	.420	.067	0.446	.646	.039	1.66	.213	.131	2.42	.148	.180	0.266	.769	.024
EL	0.929	2, 22	.410	.078	0.036	.964	.181	0.391	.681	.034	0.326	.580	.029	1.37	.276	.110
WR	0.625	2, 22	.545	.054	0.657	.528	.056	1.02	.377	.085	1.44	.255	.116	0.692	.511	.059
TH	0.190	2, 22	.828	.017	0.823	.452	.070	0.048	.953	.004	2.20	.166	.167	1.96	.165	.151
IN	0.115	2, 22	.892	.010	0.680	.517	.058	0.697	.509	.060	6.86	.024	.384	2.58	.099	.190
MI	0.383	2, 22	.686	.034	0.408	.670	.036	0.198	.822	.018	0.588	.459	.051	1.28	.297	.104
RI	0.666	2, 22	.524	.057	0.762	.478	.065	0.191	.827	.017	2.34	.154	.176	1.49	.246	.120
LI	0.156	2, 22	.857	.014	1.05	.266	.087	0.074	.929	.007	0.142	.714	.013	2.19	.136	.166

Table 4: Bonferroni-corrected paired t-tests for elbow site*effector interaction

Bonferroni-corrected alpha cutoff = .025.

Variable	Mean(±SE) values						Paired t-tests											
	Hand			Finger			Hand						Finger					
							PMv – Vertex		PMd – Vertex		PMv – PMd		PMv – Vertex		PMd – Vertex		PMv – PMd	
	PMv	PMd	Vertex	PMv	PMd	Vertex	t (11)	p	t (11)	p	t (11)	p	t (11)	p	t (11)	p	t (11)	p
PA (cm/s^2)	236 (17.4)	229 (11.8)	224 (13.3)	159 (11.6)	167 (7.76)	157 (6.89)	1.03	.324	0.548	.595	0.868	.404	0.189	.853	1.523	.156	-1.10	.295
PV (cm/s)	47.2 (3.23)	44.0 (2.61)	45.4 (2.47)	25.2 (1.63)	26.2 (1.35)	26.0 (1.30)	0.704	.496	-0.845	.416	1.35	.205	-0.671	.516	0.140	.891	-0.721	.486
PD (cm/s^2)	-181 (14.7)	-174 (10.3)	-177 (9.43)	-135 (11.6)	-140 (6.79)	-129 (4.72)	-0.442	.667	0.372	.717	-0.858	.409	-0.710	.492	-2.01	.070	0.633	.539

Supplementary figures

Figure 1: Resampled mean velocity curve for the imitator thumb comparing PMv and PMd

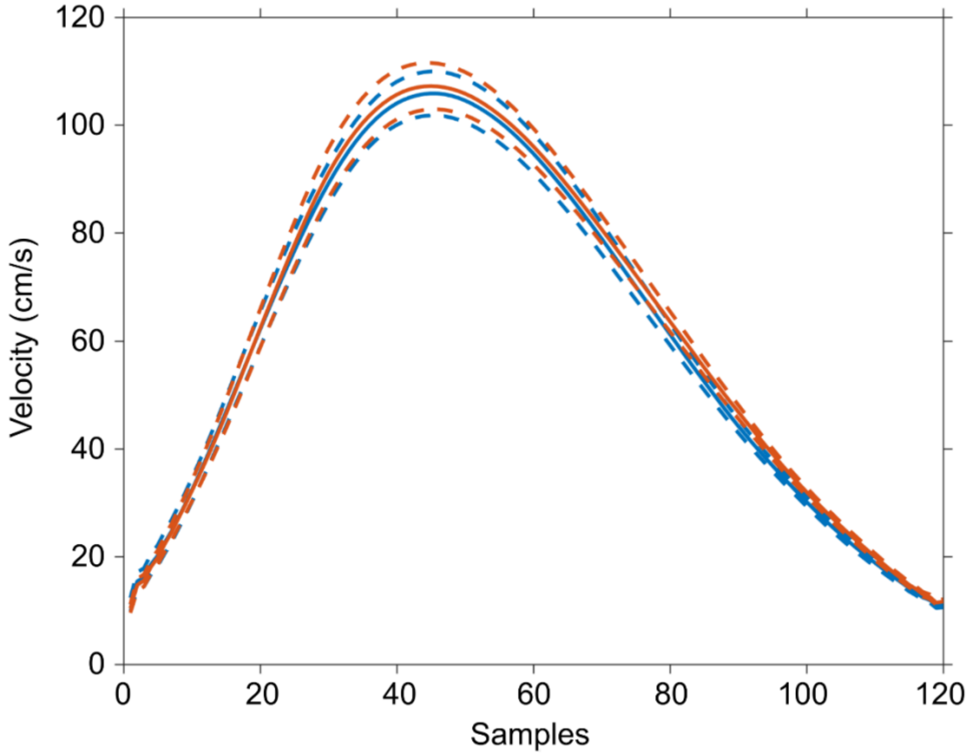

Blue = PMv, red = PMd, Dashed lines = SE.

Figure 2: Resampled mean velocity curve for the imitator index finger comparing observation and imitation stimulation over the PMv

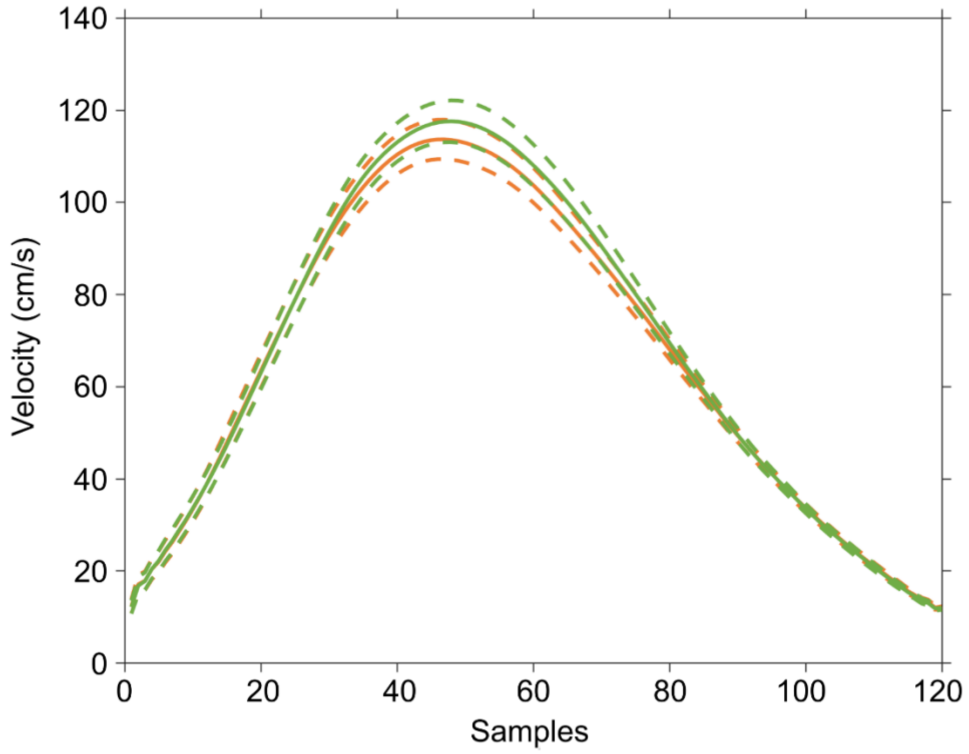

Orange = observation, green = imitation. Dashed lines = SE.

Figure 3: t-statistic plots for stimulation-site related interactions

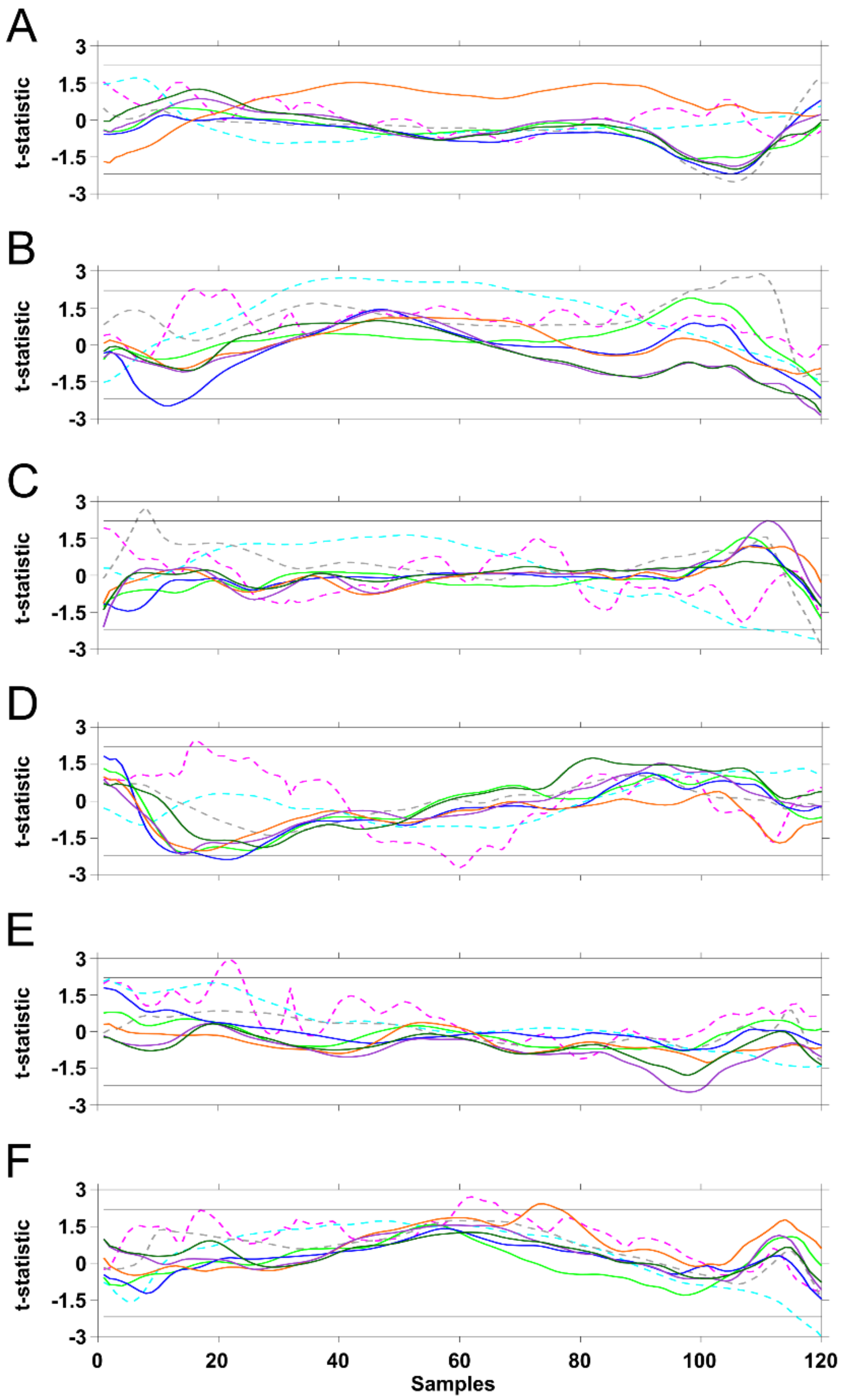

A) site*meaning B) site*effector C) site*time*meaning D) site*time*effector E) site*meaning*effector F) site*time*meaning*effector; Horizontal lines indicate positive and negative critical t-values; Dashed magenta = shoulder, dashed cyan = elbow, dashed grey = wrist, light green = thumb, blue = index finger, orange = middle finger, purple = ring finger, dark green = little finger.
